# MicroRNA-34a Mediates the Aldosterone-Induced Acceleration of Endothelial Senescence

**DOI:** 10.1155/ijhy/2339598

**Published:** 2025-02-26

**Authors:** Minyue Jia, Liya Lin, Boyun Yang, Hanxiao Yu, Shan Zhong, Xiaohong Xu, Xiaoxiao Song

**Affiliations:** ^1^Department of Ultrasonography, The Second Affiliated Hospital, Zhejiang University School of Medicine, Hangzhou, Zhejiang, China; ^2^Clinical Research Center, The Second Affiliated Hospital, Zhejiang University School of Medicine, Hangzhou, Zhejiang, China; ^3^Department of Allergy, The Second Affiliated Hospital, Zhejiang University School of Medicine, Hangzhou, Zhejiang, China; ^4^Department of Endocrinology, The Second Affiliated Hospital, Zhejiang University School of Medicine, Hangzhou, Zhejiang, China

**Keywords:** aldosterone, cellular senescence, endothelial dysfunction, microRNA-34a, NOTCH1

## Abstract

Inappropriate aldosterone production relative to sodium status is known to induce arterial hypertension and cause detrimental effects on endothelium and vascular remodeling. This study investigated whether microRNAs (miRs) serve as key mediators of aldosterone's effects on endothelial dysfunction. Using human umbilical vein endothelial cells (HUVECs) as a model system, we demonstrated that aldosterone treatment suppressed cellular proliferation and migration while promoting senescence. Mechanistically, we observed that aldosterone exposure significantly upregulated miR-34a expression in HUVECs. The functional significance of miR-34a was confirmed when specific inhibitors reversed aldosterone's antiproliferative and prosenescence effects. To elucidate the underlying molecular pathway, we performed comprehensive biological analyses, which revealed that miR-34a target genes were predominantly associated with the Notch signaling pathway. Western blot analysis further validated that miR-34a promotes senescence in HUVECs through negative regulation of NOTCH1. Collectively, our findings identify miR-34a as a crucial mediator of aldosterone-induced endothelial cell senescence via the NOTCH1 signaling pathway, suggesting its potential as a therapeutic target for aldosterone-related vascular diseases.

## 1. Introduction

Primary aldosteronism (PA) represents the most common and treatable form of secondary hypertension, with prevalence rates varying from 0.7% to 29.8%, contingent upon population selection and diagnostic criteria [1]. Compared to essential hypertension (EH), the excess aldosterone (ALD) production in PA is associated with a higher incidence of cardiovascular complications, including coronary artery disease, myocardial infarction, stroke, transient ischemic attack, atrial fibrillation, and heart failure [2]. Growing evidence demonstrates that ALD significantly impacts endothelial function through multiple mechanisms: It modulates vascular permeability and nitric oxide synthase activity [3, 4], inhibits morphogenesis and angiogenesis [5], and increases cellular stiffness [6].

Cellular senescence is an important cause of vascular endothelial dysfunction. It refers to a state of stable cell cycle arrest in which proliferating cells become resistant to growth-promoting stimuli, typically in response to DNA damage. It is mainly manifested in enhanced β-galactosidase activity, decreased cell proliferation ability, cell-cycle arrest, decreased telomerase activity, secretion of a diverse array of signaling molecules termed the senescence-associated secretory phenotype (SASP), and increased expression of senescence-related genes, such as P21, P16, E2F7, BTG2, and SULF2 [7, 8]. Recently, studies have shown that ALD promoted endothelial progenitor cell (EPC) senescence and inhibited EPC proliferation by downregulating SIRT1 [9]. Also, hyperaldosteronism can mediate the senescence of renal proximal tubule cells by mineralocorticoid receptor and p21-dependent pathways [10]. This suggests that under certain conditions ALD promotes cellular senescence. So exploring whether cellular senescence plays an important role in the process of vascular endothelial cell injury induced by ALD will help to further reveal the pathogenesis of PA and bring new ideas for its diagnosis and treatment.

MicroRNAs (miRNAs) are a type of endogenous small noncoding RNA containing 20–24 nucleotides that can specifically bind the 3′-untranslated region of target gene mRNA by base pairing, causing degradation of the target mRNA or inhibition of its translation, therefore silencing the target gene at the posttranscriptional level [11]. It has been reported that miRNAs play a very significant role in endothelial cell functions such as proliferation, migration, blood vessel formation, and apoptosis [12]. Multiple scientific studies have reported that perturbed expression of microRNA-34a-5p (miR-34a) is evident in several human pathologies, including cancers [13, 14] and cardiovascular diseases [15–17]. Specifically, miR-34a has been shown to increase the risk of congenital heart disease by modulating the Notch signaling pathway [18], and its function has been implicated in vascular senescence [19, 20], mitochondrial dysfunction [15], oxidative stress [21, 22], apoptosis [13, 23], and remodeling of multifunctional signaling pathways [24]. To our knowledge, investigations exploring the involvement of miRNAs in regulating endothelial function during elevated ALD levels remain limited.

Herein, we assessed the effects of aldosterone on proliferation, migration, and senescence of human umbilical vein endothelial cells (HUVECs) grown in vitro. Simultaneously, we screened for miRNAs whose expression levels were significantly altered in HUVECs treated with ALD for an effective duration. Then, the mechanisms of miR-34a in ALD-induced HUVEC senescence were further explored by bioinformatics analyses and in vitro validation by western blot. We expect that this study will ultimately provide new targets for the prevention and treatment of PA.

## 2. Materials and Methods

### 2.1. Cell Culture and Chemicals

HUVECs were purchased from the American Type Culture Collection (No. CRL-1730; ATCC, USA). Cells were grown in RPMI-1640 medium supplemented with 10% (V/V) denatured fetal bovine serum (FBS) and incubated at 37°C in 5% CO_2_ in compressed air. Passages 5 to 10 were used in experiments. Aldosteronism was bought from Sigma Chemical (St. Louis, MO).

### 2.2. MTS Cell Proliferation Assay

HUVECs were randomly divided into five groups. The control group consisted of cells cultured in 1640 medium containing 10% FBS without any drugs or other interventions. The four ALD-treatment groups consisted of cells cultured in 1640 medium containing 10% FBS and supplemented with the respective concentrations of ALD (100, 10, 1, or 0.1 nmol/L) for 96 h. Then, cellular proliferation was analyzed using MTS assay kit (Promega, Madison, WI) in accordance with the manufacturer's protocol. All assays were performed in triplicate wells.

### 2.3. Senescence-Associated β-Galactosidase (SA-β-Gal) Staining

HUVECs were cultured in fresh medium or in the presence of aldosteronism (10 nmol/L) for 96 h. Senescent cells were analyzed using a SA-β-gal staining kit (Beyotime Institute of Biotechnology, Shanghai, China) according to the manufacturer's instructions. The percentage of SA-β-gal-positive cells was calculated by counting the cells in 5 random fields (at least 100 cells) using bright-field microscopy, and the dark blue granules developed within the cytoplasms were considered positive for the β-galactosidase staining, suggesting senescence of the observed cells.

### 2.4. Telomerase Activity Assessment

Telomerase activity was quantitatively measured using the TeloTAGGG Telomerase PCR ELISAPLUS system (Roche, Basel, Switzerland), implementing the telomeric repeat application protocol (TRAP) assay protocol according to manufacturer's specifications.

### 2.5. Scratch or Wound Assay

HUVECs were pretreated with 10 nmol/L ALD for 48 h, and then two cross scratches were made using sterile 200-μl pipette tip (Eppendorf). Adherent cells were continued cultured with serum-free medium containing 10 nmol/L ALD for 48 h. At 0 h, 24 h, and 48 h after the scratches were generated, images were recorded using inverted microscope. Results of wound healing assay were analyzed using TScratch software that was developed by the Koumoutsakos group (CSE Lab), at ETH Zürich [25].

### 2.6. miRNA Expression Analysis Following ALD Treatment in HUVECs

#### 2.6.1. Selection of miRs for Screening in the Study

A PubMed search using the MeSH terms “endothelial cells” and “microRNAs” identified 28 miRs and one miR cluster associated with endothelial function [26]. From these, eight miRs were chosen for further experimental investigation based on their potential roles in regulating endothelial cell activity. The selected miRs were miR-21, miR-19a, miR-17-5p, miR-126, miR-320a, miR-31, miR-34a, and miR-130a.

#### 2.6.2. RNA Quantification Through Real-Time PCR Analysis (qRT-PCR)

Following established protocols, RNA isolation was conducted using the TRIzol reagent (Invitrogen, Carlsbad, CA). For the synthesis of cDNA, we utilized the miScript II RT Kit (Qiagen, Hilden, Germany), processing 1 μg of isolated RNA in accordance with the supplier's guidelines. The primer sequences corresponding to the eight miRNAs targets are detailed in Table 1. Quantitative analysis was executed on an ABI StepOnePlus platform (Applied Biosystems, CA, USA) employing miScript SYBR Green PCR Kit (Qiagen, Hilden, Germany), adhering to the recommended experimental procedures. For normalization of miRNAs expression levels, RNU6B served as the internal reference control. Fold change of relative mRNA expression was calculated using the 2^−ΔΔCt^ method, and experiments were performed in triplicate.

### 2.7. Investigation of miR-34a in ALD-Induced Senescence of HUVECs

#### 2.7.1. RNA Interference Methodology

Using Lipofectamine 2000 transfection reagent (Invitrogen, Grand Island, NY), we introduced miR-34 inhibitors (Ribobio, Guangzhou, China) into HUVEC cultures. The final inhibitor concentration was maintained at 100 nmol/L throughout the experimental procedure. And miR-34a inhibitor negative control (miR-34a inhibitor NC) as well as Lipofectamine 2000 reagent only was used as the negative control. The culture conditions involved a 6-h treatment period with small RNA complexes, after which the growth medium underwent replacement.

#### 2.7.2. ALD Treatment After Transfection

qRT-PCR was used to verify the decrease of miR-34a level after transfection with or without ALD. To compare the effects of different treatments on HUVEC senescence, the cells were divided into four groups: control group, ALD treatment only, miR-34a inhibitor + ALD, and miR-34a inhibitor NC + ALD. Then, the cell survival rate, the number of senescent HUVECs, and telomerase activity were measured after 96 h. The specific methods were the same as above.

### 2.8. Bioinformatics Analyses

#### 2.8.1. Prediction of miRNA Target Genes

Multiple bioinformatic resources were employed for miRNA target prediction, including miRTarBase, TarBase V.8, PolymiRTS 3.0, miRecord, TargetScan7.2, RNA22 2.0, PicTar-vet, miRDB, miRWalk 3.0, and miRSystem [27]. Among these, four databases (miRTarBase, TarBase V.8, PolymiRTs 3.0, and miRecord) contained experimentally validated target genes. To ensure prediction reliability, we selected only genes identified by six or more distinct databases for subsequent analysis. We then retrieved endothelial cell-associated genes from the NCBI Gene database(https://www.ncbi.nlm.nih.gov/gene/) using the search parameters “endothelial cell” AND “*Homo sapiens*”.. Finally, we identified genes showing concurrent involvement in both miRNA pathways and endothelial cellular functions for further investigation.

#### 2.8.2. Functional Analysis for Promising Target Genes

Functional annotation of the aggregated miRNA target genes was performed using the Database for Annotation, V, and Integrated Discovery (DAVID V.6.8; https://david.ncifcrf.gov/summary.jsp), encompassing both Kyoto Encyclopedia of Genes and Genomes (KEGG) pathway mapping and gene ontology (GO) enrichment analysis (*p* < 0.05). To establish functional relationships among putative miR-34a target genes, we constructed a protein–protein interaction (PPI) network using STRING database V.11.0 (https://string-db.org/). The network analysis was conducted using Cytoscape 3.8.0, an open-source bioinformatics platform, where hub genes were identified through the cytoHubba plugin based on STRING-derived network data. For comprehensive functional characterization, we employed ClueGO and CluePedia plugins to analyze pathway associations and functional annotations, enabling detailed examination of GO terms and KEGG pathway enrichment patterns among significant genes.

### 2.9. In Vitro Validation

#### 2.9.1. Cell Processing

HUVECs were divided into six groups for experimentation, including a control group, ALD treatment only, miR-34a mimic (Ribobio, Guangzhou, China) + ALD, miR-34a mimic negative control (miR-34a mimic NC; Ribobio, Guangzhou, China) + ALD, miR-34a inhibitor + ALD, and miR-34a inhibitor NC + ALD. ALD treatment was defined as exposure to 10 nmol/L of ALD for a duration of 96 h.

#### 2.9.2. Cell-Cycle Analysis by Flow Cytometry

HUVEC cells (2 × 10^5^/well) were seeded into 6-well plates and then centrifuged, washed twice with cold PBS, and fixed with 70% precooled ethanol overnight at 4°C. The fixed cells were subsequently washed with cold PBS and stained with PI (50 μg/mL), RNase A (100 μg/mL), and 0.2% Triton-X for 30 min at 4°C in the dark. Finally, the stained cells were analyzed using a flow cytometer (FCM; Beckman). The distribution of cells in the G1, S, and G2/M phases of the cell cycle was determined based on their DNA content using ModFit LT Mac 3.3 software (BD Biosciences).

#### 2.9.3. SASP Detection

The SASP constitutes a hallmark of senescent cells and mediates many of their pathophysiological effects [28]. Consequently, the mRNA expression levels of IL-6 and IL-8, as pivotal constituents of this phenomenon, were quantified via qRT-PCR. Total RNA extraction from the designated cell populations was executed utilizing TRIzol reagent (Takara). Subsequently, 1.5 μg of RNA served as the template for complementary DNA (cDNA) synthesis, adhering to the manufacturer's protocols (Promega). Following cDNA synthesis, qRT-PCR assays targeting IL-6 and IL-8 were conducted, consistent with established methodologies [29, 30].

#### 2.9.4. Western Blots and Antibodies

For total protein extraction, samples underwent cell lysis using a mixture of RIPA buffer (200 μL) combined with PMSF (1 mM) while maintained on ice for 5 min. Protein samples (30 μg) were loaded per well and underwent separation through 10% SDS-PAGE. The separated proteins were subsequently transferred onto PVDF membranes obtained from Bio-Rad (Hercules, CA) via electroblotting. The membranes underwent blocking treatment with 1x TBST containing 5% milk solution, followed by overnight exposure to primary antibodies at 4°C. Subsequently, the samples were treated with secondary antibodies for a duration of 1 hour. Visualized with the enhanced chemiluminescence (ECL), images were obtained with a chemiluminescence imager (Amersham ImageQuant 800, USA). All antibodies were as follows: anti-p21 (1:1000, CST, no. 2947S), anti-p16 (1:1000, CST, no92803S), anti-NOTCH1 (1:1000, CST, no. 4380), anti-β-tubulin (1:1000, CST, no. 2148S), and anti-rabbit IgG, HRP-linked (1:5000, CST, no. 7074). The β-tubulin was used as an internal control. Protein expression levels were semiquantified using ImageJ software (Version 4.62; National Institutes of Health).

### 2.10. Statistical Analysis

Data were presented as the means ± SEM. ANOVA was used if the groups were consistent with normal distribution and homogeneity of variance, and Student–Newman–Keuls (SNK) was used for pairwise comparison. If not, the Kruskal–Wallis rank sum test is used. Each experimental procedure was conducted in triplicate at minimum, and statistical significance was defined as *p* < 0.05. Data management and analyses were performed using SAS (SAS 9.1; SAS Institute, Cary, NC).

## 3. Results

### 3.1. Effects of ALD on HUVEC Proliferation

**Figure 1:**
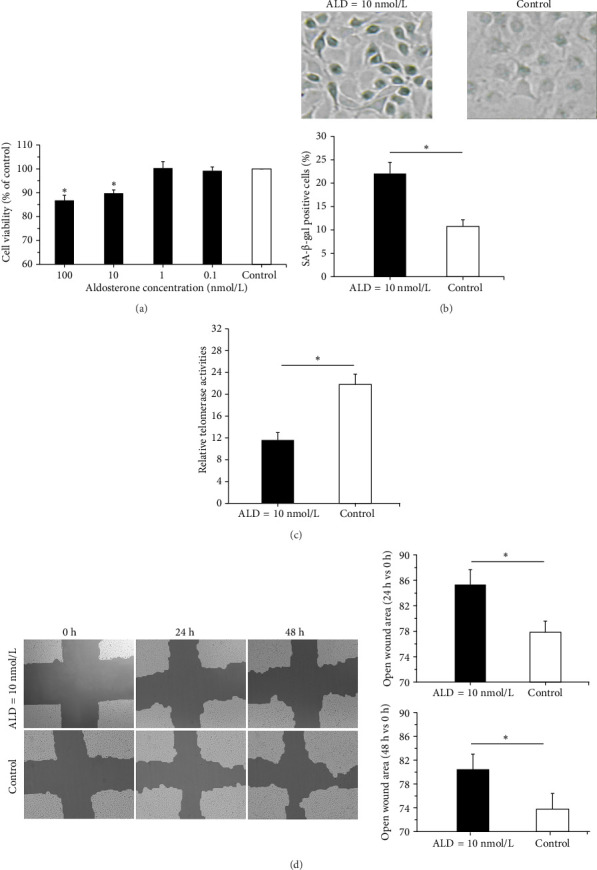
Effects of aldosterone on HUVEC proliferation, senescence, and migration. (a) Impact of aldosterone on HUVEC proliferation. HUVECs were treated with varying concentrations of aldosterone for 96 h, with untreated cells serving as controls. Aldosterone (100 nmol/L, 10 nmol/L) significantly inhibited HUVECs proliferation compared to controls. (b) Influence of aldosterone on β-gal staining. Following coculture of HUVECs with 10 nmol/L aldosterone for 96 h, β-gal staining identified senescent cells, evidenced by blue cytoplasmic staining. Aldosterone notably promoted HUVECs senescence compared to the control group. (c) Effect of aldosterone on telomerase activity. Telomerase activity in HUVECs was markedly reduced after 96 h of treatment with 10 nmol/L aldosterone, as determined by the telomeric repeat amplification protocol (TRAP) assay, in comparison to the control group. (d) Impact of aldosterone on HUVECs migration. In vitro wound healing assays revealed that aldosterone (10 nmol/L) hindered HUVEC migration. HUVECs were pretreated with 10 nmol/L aldosterone for 48 h, followed by creating two perpendicular scratches using a sterile 200 μL pipette tip (Eppendorf). Adherent cells were further cultured with serum-free medium containing 10 nmol/L aldosterone for 48 h. Images were captured at 0, 24, and 48 h postscratch generation using an inverted microscope. ⁣^∗^*p* < 0.05 compared to control. All assays were performed in triplicate.

In order to evaluate the proliferating potential of HUVECs exposed to different concentrations of ALD (100, 10, 1, 0.1 nmol/L), the MTS assay was carried out. When ALD concentrations increased, HUVEC survival decreased. Statistical results showed that 96-h intervention with ALD (100 nmol/L, 10 nmol/L) significantly inhibited the proliferation of HUVECs (*p* < 0.05), although the cell survival rate of the 100 nmol/L ALD group is lower than that of the 10 nmol/L ALD group, there was no statistical difference (Figure 1(a)).

### 3.2. Effects of ALD on Cellular Senescence of HUVECs

#### 3.2.1. SA-β-Gal Staining

β-Gal staining is one of the few widely accepted biological signs of cell senescence. It is a classic method for identifying senescent cells. HUVECs treated with ALD (10 nmol/L) showed senescence characteristics that include increased cell volume, slowed growth, a flattened shape, and emergence of more granules and nuclei after 96 h. At the same time, compared with the normal control group, ALD-treated cells had more β-gal staining positive cells (*p* < 0.05, Figure 1(b)).

#### 3.2.2. Telomerase Activity Assay

Telomerase is a DNA polymerase that uses its own RNA as a template. It delays cell senescence by maintaining the length of telomeres on the ends of chromosomes during mitosis. Therefore, its activity has an important effect on cell senescence. After 96 h of 10 nmol/L ALD treatment, the relative telomerase activity of the HUVECs was 11.6 ± 1.4, whereas the relative telomerase activity of the control cells was 21.8 ± 1.9 (*p* < 0.05). Therefore, ALD inhibited telomerase activity of HUVECs and promoted cell senescence (Figure 1(c)).

### 3.3. Effects of ALD on HUVEC Migration

We used cell scratch assay to determine the effects of ALD on migration of HUVECs. The results showed that compared with the control group, ALD inhibited migration of HUVECs (*p* < 0.05, Figure 1(d)).

### 3.4. Effects of ALD on miRNA Expression of HUVECs

**Figure 2:**
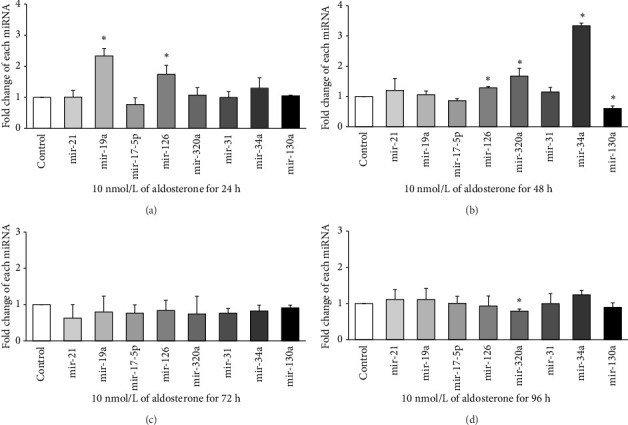
Expression analysis of endothelial cell-associated microRNAs in aldosterone-treated HUVECs. Quantitative PCR measurements were performed to assess microRNA levels at 24, 48, 72, and 96 h following exposure to aldosterone (10 nmol/L). A significant upregulation of miR-34a was observed at the 48-h time point postaldosterone treatment. ⁣^∗^*p* < 0.05 versus untreated controls.

Expression of eight miRNAs (miR-21, miR-19a, miR-17-5p, miR-126, miR-320a, miR-31, miR-34a, and miR-130a) with known vascular endothelial function was evaluated in HUVECs treated with 10 nmol/L ALD for 24, 48, 72, or 96 h, using qRT-PCR. At 24 h (Figure 2(a)), significant upregulation was observed for miR-19a and miR-126, with approximately 2.33-fold and 1.74-fold increases, respectively, compared to the control (*p* < 0.05). After 48 h of ALD treatment (Figure 2(b)), miR-34a showed the most pronounced increase, with a fold change of about 3.34 compared to the control (*p* < 0.05). In addition, miR-126 and miR-320a were significantly upregulated at this time point, although miR-130a displayed a notable decrease. At 72 h (Figure 2(c)), no significant changes were observed in the expression of any of the miRNAs examined. By 96 h (Figure 2(d)), most miRNA levels had returned to baseline, with only miR-320a showing a significant decrease compared to the control (*p* < 0.05). These results suggest a dynamic and time-dependent regulation of miRNAs expression in response to ALD treatment, with the most pronounced changes occurring at 24 and 48 h posttreatment.

### 3.5. Role of miR-34a in ALD-Induced Senescence of HUVECs

#### 3.5.1. miR-34a Expression Downregulated After miR-34a Inhibitor Transfection

**Figure 3:**
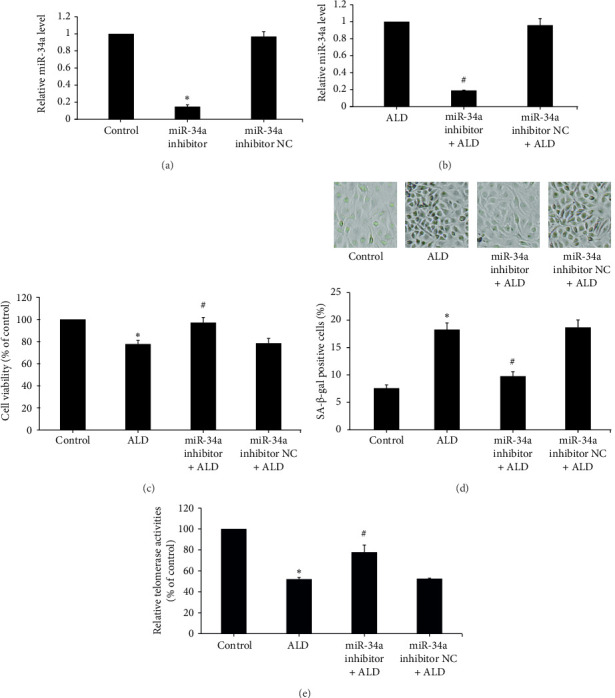
miR-34a inhibition reversed the aldosterone-mediated inhibition of endothelial cell proliferation and promotion of senescence. (a, b) After miR-34a inhibitor transfection, miR-34a RNA levels were significantly reduced after 48 h with or without aldosterone treatment. (c) The cell survival rate of miR-34a inhibitor group was significantly higher than that of the ALD group. MTS test was used to detect cell proliferation with aldosterone at 96 h. (d) After miR-34a inhibitor transfection, the number of senescent HUVECs is significantly reduced compared to the ALD group. SA-β-gal staining was used to identify senescent cells with aldosterone at 96 h. (e) Telomerase activity in the miR-34a inhibitor group was significantly higher than that of the ALD group. TRAP-ELISA method was used to detect telomerase activity with aldosterone at 96 h. ⁣^∗^*p* < 0.05 compared to control. ^#^*p* < 0.05 compared to ALD group. All assays were performed in triplicate.

After miR-34a inhibitor transfection, miR-34a levels were significantly reduced with or without ALD, while inhibitor NC was not significantly different from the control group or ALD group (Figures 3(a) and 3(b)).

#### 3.5.2. Inhibition of miR-34a Abrogated ALD-Mediated Inhibition of Endothelial Proliferation

miR-34a inhibitor was employed to reduce the level of HUVEC miR-34a, and then the effects of aldosterone on the proliferation of HUVECs in this situation were investigated. MTS assay results showed that the cell survival rate of the miR-34a inhibitor group was statistically increased compared to the ALD group (*p* < 0.05). While ALD negatively affected the multiplication of HUVECs, subsequent transfection with miR-34a inhibitor successfully reversed these growth-limiting effects (Figure 3(c)).

#### 3.5.3. Reducing miR-34a Levels Prevented ALD-Mediated Enhancement of Senescence in Endothelial Cells

HUVECs were transfected with the miR-34a inhibitor and then treated with 10 mmol/L of ALD for 96 h. The number and proportion of senescent HUVECs of the miR-34a inhibitor were significantly lower than that of the ALD group (9.8 ± 0.8% vs. 18.3 ± 1.2%, *p* < 0.05, Figure 3(d)). And the relative telomerase activity of the miR-34a inhibitor was significantly higher than that of the ALD group (*p* < 0.05, Figure 3(e)).

### 3.6. Identification of miR-34a Target Genes and Bioinformatics Analysis

Through comprehensive analysis across 10 distinct target gene prediction databases and the Gene repository, we identified 51 potential genes regulated by miR-34a (Additional file 1).

**Figure 4:**
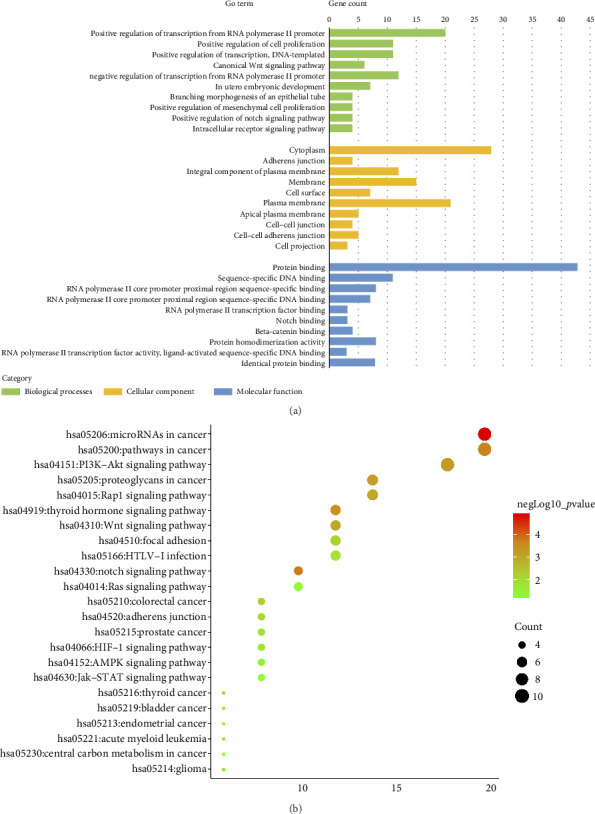
Results of GO enrichment and KEGG pathway analysis using DAVID. (a) The top 10 most significantly enriched GO terms. (b) Enriched KEGG pathways of 51 overlapping target genes of miR-34a.

Our functional analysis using DAVID revealed a complex regulatory network involving these target genes. This network encompassed 23 distinct KEGG pathways and multiple GO classifications, specifically: 114 biological process terms, 14 cellular component categories, and 25 molecular function classifications. The top 10 GO terms and KEGG pathways are presented in Figure 4. The results indicate that the target genes were significantly enriched in the cytoplasm and demonstrated key molecular functions, including protein binding, DNA binding, transcriptional activator activity, and Notch binding.

**Figure 5:**
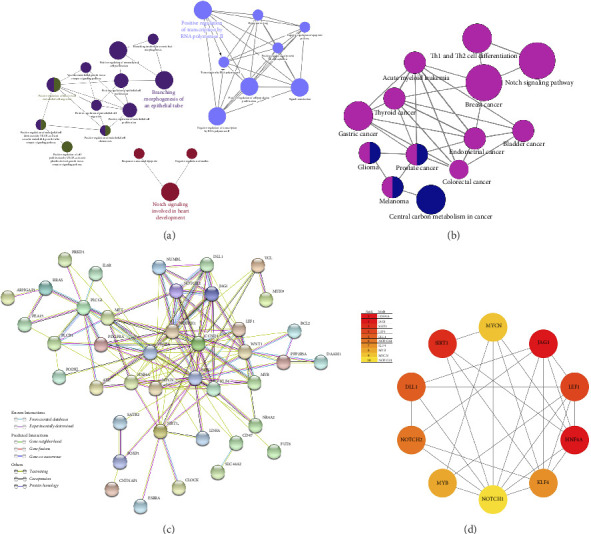
Detailed insights into the functional roles and interactions of miR-34a target genes as elucidated through computational analysis. (a) The mapped networks of enriched biological processes of target genes constructed using Cytoscape software. The size of the circle represents the level of statistical significance (the smaller the *p* value, the larger the circle), and different colors indicate different groups. (b) The mapped networks of KEGG pathways analysis using Cytoscape software. The size of the circle represents the level of statistical significance (the smaller the *p* value, the larger the circle), and different colors indicate different groups. (c) Protein–protein interaction networks of the 51 overlapped target genes of miR-34a (hide disconnected nodes). The balls represent the gene nodes, and the connecting lines represent the interactions between genes. Line colors represent the evidence of PPI. (d) The top 10 hub target genes of miR-34a with cytoHubba in the PPI network. The color of the nodes reflects the importance of the target (the darker the color, the more important the target).

Subsequent Cytoscape analysis identified three primary biological processes where these target genes were concentrated: regulation of transcription from RNA polymerase II promoter, branching morphogenesis of epithelial tubes, and Notch signaling in heart development (Figure 5(a)). Notably, KEGG pathway analysis emphasized the significant involvement of miR-34a target genes in the Notch signaling pathway (*p*=0.000129) and various cancer-associated pathways (Figure 5(b)).

To further characterize the relationships among these genes, we performed PPI network analysis, which unveiled 130 distinct interactions within the 51 target genes (Figure 5(c)). Subsequently, using the DMNC method in cytoHubba, we identified 10 hub genes: HNF4A, JAG1, SIRT1, LEF1, DLL1, NOTCH2, KLF4, MYB, MYCN, and NOTCH1 (Figure 5(d)). These genes were all negatively regulated by miR-34a, as confirmed by previous studies.

In summary, the bioinformatics analysis of miR-34a target genes reveals that the Notch signaling pathway is significantly enriched and plays a crucial role in miR-34a-mediated regulation of endothelial cell function. Notably, key hub genes such as JAG1, DLL1, NOTCH1, and NOTCH2, which are negatively regulated by miR-34a, are involved in this pathway.

### 3.7. In Vitro Validation Results

**Figure 6:**
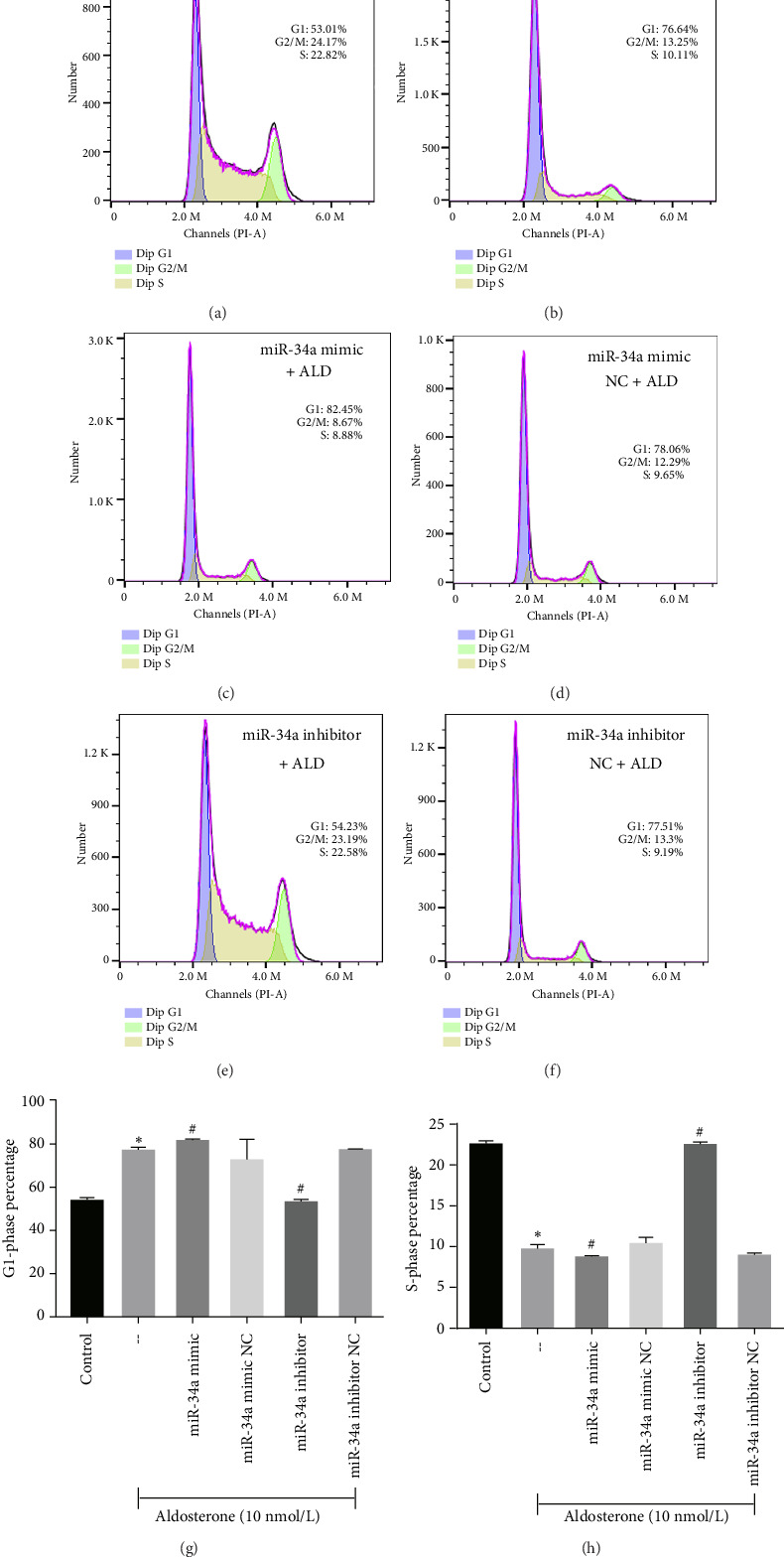
Cell cycle analysis of HUVECs after ALD treatment with miR-34a modulation. Cell cycle distribution was obtained by PI staining and flow cytometry analysis in HUVEC cells following treatment with 10 nmol/L of ALD for 96 h. Results indicate that ALD induces cell cycle arrest, which is mitigated by the inhibition of miR-34a. ⁣^∗^*p* < 0.05 compared to control. ^#^*p* < 0.05 compared to -- group (aldosterone treatment only).

To elucidate the underlying mechanisms associated with the observed reduction in cell proliferation rates, we conducted cell cycle analysis on HUVEC cells. Analysis via flow cytometry revealed that, upon exposure to 10 nmol/L ALD for 96 h, a marked increase in the proportion of HUVEC cells in the G1 phase and a concomitant decrease in the S phase were observed when compared to the untreated control group. Notably, elevated levels of miR-34a prompted cell cycle arrest, effectively impeding cellular proliferation. Conversely, the suppression of miR-34a mitigated this regulatory effect (Figure 6).

**Figure 7:**
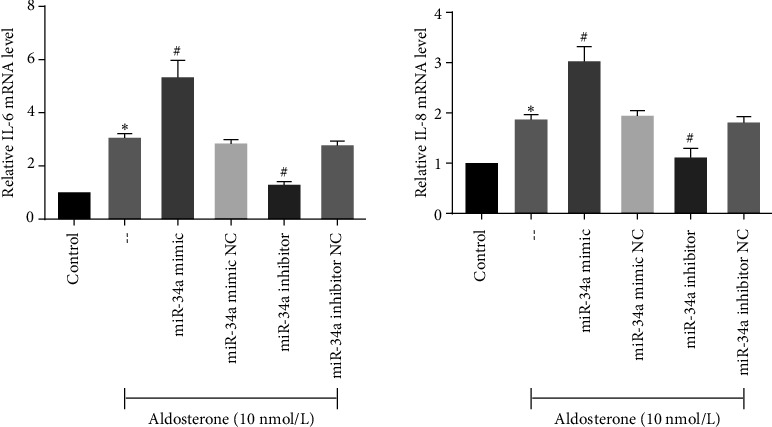
Transcript-level expression of IL-6 and IL-8 posttransfection in HUVECs. ALD increased the IL-6 and IL-8 transcript-level expression, while miR-34a inhibitors attenuated it. Cell lysates were used to detect IL-6 and IL-8 mRNA levels by qRT-PCR. Results were normalized to controls, and histograms represent the relative intensity of IL-6 and IL-8 levels. ⁣^∗^*p* < 0.05 compared to control. ^#^*p* < 0.05 compared to -- group (aldosterone treatment only).

The SASP is instrumental in facilitating cell-autonomous functions such as senescence-associated growth arrest. Treatment with ALD was found to augment the transcript-level expression of pivotal SASP components, including IL-6 and IL-8, in HUVECs. In stark contrast, the inhibition of miR-34a led to a decline in the transcript-level expression of these SASP components in ALD-treated HUVECs (Figure 7).

**Figure 8:**
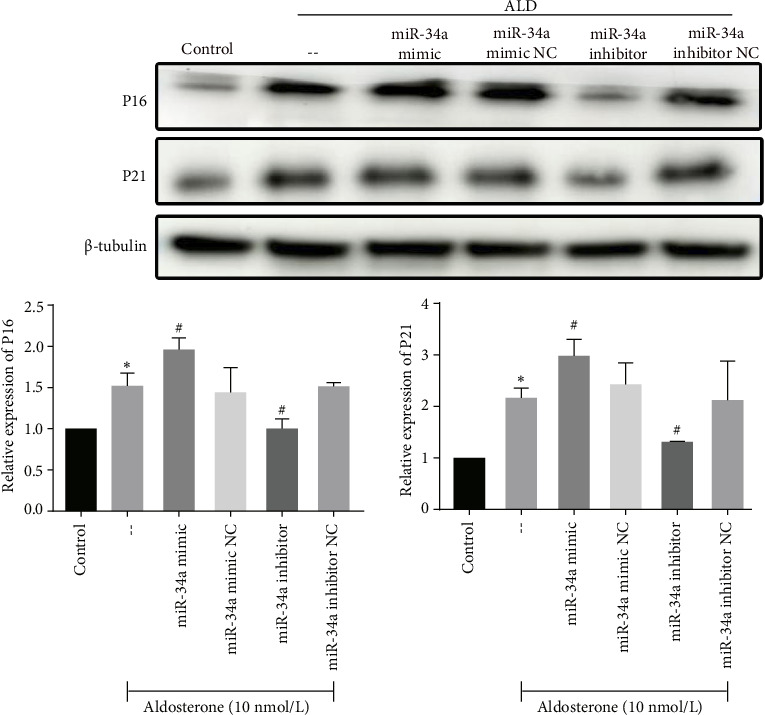
Western blot analysis of P21 and P16 protein expression in HUVECs after transfection. ALD treatment resulted in a notable upregulation of the P21 and P16 protein level. miR-34a mimics significantly increased the protein expression levels of P21 and P16, while miR-34a inhibitors attenuated them in HUVECs. ⁣^∗^*p* < 0.05 compared to control. ^#^*p* < 0.05 compared to -- group (aldosterone treatment only).

In alignment with these findings, the expression levels of P21 and P16, markers indicative of cellular senescence, were significantly elevated in response to ALD treatment. This effect was further enhanced by miR-34a mimics, whereas miR-34a inhibitors diminished it (Figure 8).

**Figure 9:**
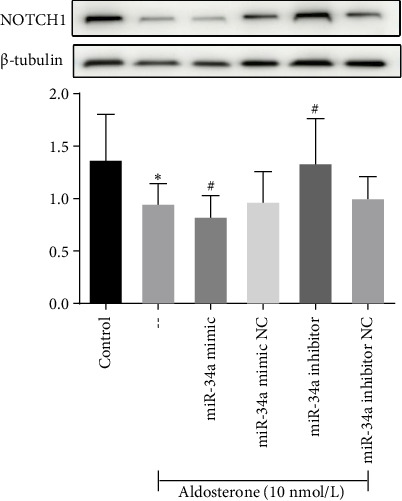
Western blot analysis of NOTCH1 protein expression in HUVECs following miR-34a modulation. NOTCH1 protein levels were significantly downregulated in response to miR-34a mimic transfection, whereas miR-34a inhibitor treatment resulted in upregulation of NOTCH1 expression. ⁣^∗^*p* < 0.05 versus control group; ^#^*p* < 0.05 versus aldosterone-treated group.

Meanwhile, subsequent to 96 h of treatment with ALD, there was a notable decrease in NOTCH1 protein expression when compared to untreated controls (*p* < 0.05). Further analysis demonstrated that introducing miR-34a mimics suppressed NOTCH1 protein expression, while treatment with miR-34a inhibitors enhanced its levels (Figure 9). This finding has shed light on the senescence-promoting effect of miR-34a in HUVECs through negative regulation of NOTCH1.

## 4. Discussion

Elevated plasma ALD levels are the hallmark of PA. While ALD primarily regulates sodium retention, potassium excretion, and diuresis, chronic ALD excess induces multiple detrimental effects on the endothelium. These pathological changes include impaired vascular relaxation, enhanced oxidative stress, vessel inflammation, vascular remodeling, and accelerated atherosclerosis [2]. Endothelial dysfunction, a well-established fundamental mechanism underlying cardiovascular diseases, serves as a significant predictor of adverse cardiac events [31]. Substantial evidence demonstrates that ALD is a key mediator in the pathogenesis of endothelial dysfunction. This prompts the question: How do key vascular endothelial cell functions—such as proliferation, migration, and senescence—respond to ALD intervention? To address this query, we utilized an in vitro culture of HUVECs to directly observe the impact of ALD. HUVECs, derived from the endothelial cells of newborn umbilical cords, are the most common cell line used in vascular endothelial cell studies due to their exhibiting characteristics of vascular endothelial cells. Our study identified that ALD inhibited proliferation and migration and promoted senescence of HUVECs, providing insights into the mechanisms underlying ALD's role in the development of endothelial dysfunction and associated cardiovascular diseases.

ALD, as a mineralocorticoid, may cause a variety of cells in different tissues in the body to respond differently, which has important implications for understanding its role in various physiological and pathological processes. In the case of vascular smooth muscle cells, ALD may promote cell proliferation and hypertrophy by activating mitogen-activated protein kinase (ERK1/2, c-Jun NH2-terminal kinase, and p38) signaling pathways [32–34], thereby causing vascular remodeling and angiosclerosis [35]. In addition, ALD also promoted vascular smooth muscle cell migration via the Rho kinase signaling pathway [36]. In contrast, ALD may exert opposite effects in endothelial cells. Recent studies in animal models showed that aldosterone mediates apoptosis and antiproliferative effects in rat aortic endothelial cells by activating the G protein-coupled estrogen receptor (GPER) and then phosphorylating ERK [37]. Consistent with these findings, our study demonstrated that aldosterone inhibited proliferation and migration of HUVECs. Therefore, under different circumstances, the signaling pathway of aldosterone is very complicated, highlighting the need for further research to elucidate the diverse mechanisms underlying ALD's actions in various cell types and their contributions to health and disease.

Cellular senescence represents a progressive state characterized by irreversible cell cycle arrest and loss of proliferative capacity during repeated cellular divisions. In aged blood vessels, the accumulation of senescent vascular cells creates a dual impact: It triggers a proinflammatory microenvironment while simultaneously reducing regenerative capacity. This combination promotes vascular dysfunction and accelerates atherosclerosis development. Mechanistically, studies have demonstrated that ALD, acting through the mineralocorticoid receptor in conjunction with the AT1 receptor, activates the Ras/NF-kappaB and AP-1/P53/P21 signaling cascade. This pathway, mediated by oxidative stress, synergistically promotes vascular senescence [38]. In the current study, we discovered that ALD induces senescence in HUVECs. This was evidenced by alterations in cell morphology, an increase in β-galactosidase-staining positive cells, cell cycle arrest, secretion of SASP, and elevated expression of senescence markers P21 and P16. Furthermore, our study showed that ALD can significantly inhibit telomerase activity, preventing the maintenance of telomere length, structure, and function, thereby accelerating the process of endothelial cell senescence [39]. This is likely to be one of the reasons whereby ALD exposure leads to cellular senescence. In addition, the decline in cell proliferation is also one of the manifestations of cell senescence [40].

Further, we screened eight miRNAs that are considered to be closely related to endothelial cell function and found that miRNA-34a levels were significantly upregulated. MiR-34a has been reported to regulate cell behaviors, including cell cycle progression, apoptosis, senescence, and migration, as well as angiogenesis in p53-dependent or independent manners [41, 42]. These regulatory roles have been observed in multiple organs and cell types. For example, the expression of miR-34a was significantly increased in the brain, spleen, and heart of aged mice [20]. Furthermore, when researchers introduced miR-34a into human fibroblasts for ectopic overexpression, about 60% of the transfected cells appeared to be senescent both morphologically and molecularly [43]. Similarly, Tazawa et al. transfected miR-34a into human colon cancer HCT116 cells, RKO cells, and p53-mutated SW480 cells and found that the volume of transfected cells increased and that cells showed signs of aging and staining with the SA-β-gal, indicating that miR-34a can induce cellular senescence [44]. Building upon these findings, recent studies have emphasized the important role of miR-34a as an intermediary in the senescence and dysfunction of myocardium and endothelial cells [19, 45]. Boon et al. found that silencing or gene knockdown of miR-34a can reduce the apoptosis of senescence-associated myocardial cells, and miR-34a inhibitors can reduce apoptosis and fibrosis after acute myocardial infarction, thereby improving myocardial function recovery [46]. Moreover, miR-34a overexpression has been shown to induce senescence of endothelial cells and endothelial progenitor cells and inhibit cell proliferation by inhibiting cell cycle progression [19, 47]. Consistent with these findings, our study demonstrated that artificially downregulating the expression level of miR-34a reversed ALD-induced senescence. Taken together, these results suggest that miR-34a plays a crucial role in ALD-mediated endothelial cell senescence.

To delineate miR-34a′s molecular mechanisms, we systematically identified putative target molecules and conducted comprehensive pathway analyses to map functional interactions. Combined with the GO and KEGG analysis results of 51 endothelial cell function–related target genes, we found that the target genes of miR-34a were significantly enriched in the Notch signaling pathway, of which NOTCH2, NOTCH1, DLL1, JAG1, and NUMBL were involved. Further PPI analysis also confirmed that NOTCH2, NOTCH1, DLL1, and JAG1 were hub genes. The Notch signaling pathway represents a highly conserved molecular mechanism that orchestrates cell fate decisions and differentiation processes during tissue development [48]. In the cardiovascular system, specifically, Notch signaling plays crucial regulatory roles in multiple developmental processes, including cardiomyocyte differentiation, epithelial-to-mesenchymal transition during valvulogenesis, and vascular system development [49]. Among the four mammalian Notch receptors (NOTCH1-4) associated with the Notch pathway, NOTCH1 is a specific target of miR-34a, as confirmed by evidence from experimental investigations [50, 51]. This interaction is particularly significant as NOTCH1 serves as a crucial protective factor against atherosclerosis. Research has demonstrated that miRs-34a potentially promotes atherosclerotic development through multiple mechanisms, primarily by modifying endothelial and vascular smooth muscle cell behavior. Specifically, miR-34a triggers cellular aging processes and SASP, while simultaneously suppressing the protective effects of NOTCH1, thereby contributing to cardiovascular pathology [52]. Lastly, Li et al. demonstrated that indoxyl sulfate (IS), a uremic toxin with atherogenic properties, can significantly inhibit HUVEC viability, migration, and proliferation by upregulating miR-34a, which targets the NOTCH1 signaling pathway [53]. Given these findings, we hypothesized that miR-34a may also regulate the NOTCH1 signaling pathway during ALD-mediated cellular senescence. In vitro validation experiments by western blots confirmed this hypothesis. Our results indicated that suppression of miR-34a could increase NOTCH1 expression at protein level. Conversely, when miR-34a expression was upregulated, the protein level of NOTCH1 was decreased. Thus, together with the findings of previous research and our results, we speculated that the dysfunctional NOTCH1 was implicated in miR-34a-mediated endothelial cell senescence.

Meanwhile, studies have demonstrated that the disruption of NOTCH1 in vascular endothelial cells markedly reduces the maximum number of population doublings. This reduction is accompanied by an increase in SA-β-gal activity and upregulation of P53, P21, and P16 expression [54]. Notably, upon Notch activation, both P21, a pivotal protein in initiating senescence, and P16, another crucial factor in maintaining senescence, undergo downregulation, thereby contributing to senescence suppression. This regulatory mechanism operates through the P53–P21 and P16INK4A pathways, which control cell cycle arrest by inhibiting specific cyclin-dependent kinases (CDK2, CDK4, and CDK6). The resulting inhibition preserves retinoblastoma protein (RB) in its hypophosphorylated form, facilitating two essential cellular processes: first, the suppression of S-phase gene expression through sequestration of transcription factors (E2F, DP1, and DP2), and second, the recruitment of histone deacetylases (HDACs) to modify heterochromatin structure [55]. These findings affirm the involvement of the Notch–P53–P21 or Notch–P16 axis in regulating cell senescence. In addition to the direct effects of NOTCH1 disruption on P53–P21 and P16, the downregulation of Notch signaling also influences the SASP. Specifically, Notch signaling downregulation initiates a DNA damage response (DDR)-dependent “transitional SASP,” which is amplified by mechanistic target of rapamycin (mTOR). This process is mediated by cell surface–associated interleukin-1*α* (IL-1*α*) binding to its receptor (IL-1R), triggering either cell-autonomous IL-1*α* signaling or p38 mitogen-activated protein kinase (p38 MAPK) activation. Subsequently, nuclear factor-κB (NF-κB) activation drives the secretion of “late SASP” components, including metalloproteinases (MMPs), IL-6, and IL-8. At this juncture, IL-6 and IL-8 can further reinforce cell cycle arrest, indicating a complex interplay of signaling pathways in regulating cellular senescence [55–57].

**Figure 10:**
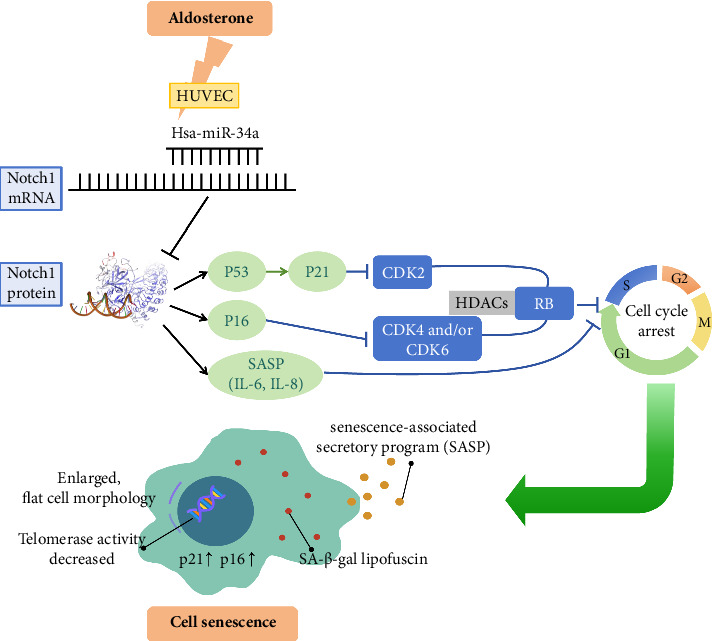
Proposed model of the microRNA-34a–NOTCH1 signaling axis in the regulation of aldosterone-induced premature senescence of endothelial cells. Schematic illustrating how aldosterone-induced miR-34a upregulation inhibits NOTCH1 signaling in HUVECs, leading to cellular changes such as altered morphology, increased SA-β-gal-positive cells, reduced telomerase activity, cell cycle arrest, SASP secretion, and heightened P21 and P16 expression. NOTCH1 inhibition elevates P21 and P16 levels, promoting CDK activity and RB hypophosphorylation and suppressing S-phase gene expression. In addition, downregulation of Notch signaling triggers a DDR-dependent “transitional SASP,” amplified by mTOR, culminating in endothelial cell senescence with a “late SASP” secretion profile comprising IL-6 and IL-8.

Based on the findings presented, we propose a potential model of the microRNA-34a–NOTCH1 signaling axis in the regulation of ALD-induced premature senescence of endothelial cells (Figure 10). This model integrates the role of miR-34a, NOTCH1, and the various signaling pathways involved in cellular senescence, providing a comprehensive understanding of the underlying mechanisms.

In addition, it is worth noting that the temporal dynamics of miR-34a expression in response to ALD treatment reveal a complex regulatory pattern. Our results demonstrate a significant upregulation of miR-34a at 48 h posttreatment, followed by a nonsignificant decrease at 96 h. This biphasic response suggests a nuanced role for miR-34a in ALD-induced endothelial senescence. The initial spike in miR-34a expression likely represents an early trigger for senescence pathways, consistent with its known function as a senescence mediator. The subsequent reduction, although not statistically significant, may indicate the activation of compensatory mechanisms or a shift in cellular response to prolonged ALD exposure. Importantly, while miR-34a upregulation peaks at 48 h, the full senescent phenotype—characterized by an increase in β-galactosidase-staining positive cells, cell cycle arrest, secretion of SASP, and elevated expression of senescence markers P21 and P16—manifests only at 96 h. This temporal disparity between peak miR-34a expression and the emergence of senescence markers underscores the complex, multistep nature of the senescence process. Our choice to focus on the 96-h time point for subsequent experiments was thus driven by the need to capture the fully developed senescent state, rather than just the initiating molecular events. This approach aligns with previous studies, such as Badi et al. [58], which have observed similar temporal patterns in other models of cellular senescence. Future investigations could elucidate the mechanisms governing this temporal regulation and its implications for the progression and maintenance of endothelial senescence, potentially revealing new targets for intervention in ALD-induced vascular aging.

## 5. Conclusions

In summary, our study identified that ALD inhibited proliferation and migration of HUVECs and promoted cellular senescence. ALD significantly upregulated the expression of miR-34a, which in turn mediated the senescence of HUVECs, as demonstrated by the inhibition of miR-34a blocking this effect. Bioinformatic analysis revealed that target genes of miR-34a were mainly involved in the Notch signaling pathway. Subsequent in vitro validation using western blot confirmed that miR-34a promotes senescence in HUVECs by negatively regulating NOTCH1. In conclusion, miR-34a may play a crucial role in ALD-mediated endothelial cell senescence through the NOTCH1 signaling pathway. This finding suggests that targeting miR-34a or the NOTCH1 signaling pathway could be a novel therapeutic approach for treating ALD-associated vascular diseases.

## Figures and Tables

**Table 1:** List of primers used in qRT-PCR.

	Sense primers (5′–3′)	Antisense primers (5′–3′)
miR-21	CGGTAGCTTATCAGACTGATGTTGA	Provided by miScript SYBR Green PCR kit (Qiagen, Hilden, Germany)
miR-19a	TGTGCAAATCTATGCAAAACTGA	
miR-17-5p	CAAAGTGCTTACAGTGCAGGTAG	
miR-126	TCGTACCGTGAGTAATAATGCG	
miR-320a	CTGGGTTGAGAGGGCGA	
miR-31	GCAAGATGCTGGCATAGCT	
miR-34a	GGCAGTGTCTTAGCTGGTTGT	
miR-130a	CAGTGCAATGTTAAAAGGGCAT	
U6	Provided by Genecopoeia (Rockville, USA)	

## Data Availability

The data that support the findings of this study are available from the corresponding author, Xiaoxiao Song, upon reasonable request.

## Nomenclature

PA: Primary aldosteronism
ALD: Aldosterone
SASP: Senescence-associated secretory phenotype
SA-β-gal: Senescence-associated β-galactosidase
EH: Essential hypertension
EPCs: Endothelial progenitor cells
HUVECs: Human umbilical vein endothelial cells
KEGG: Kyoto Encyclopedia of Genes and Genomes
GO: Gene ontology
DAVID: Database for Annotation Visualization and Integrated Discovery
PPI: Protein–protein interaction

## Supporting Information

Additional file 1: the target genes of miR-34a predicted by 10 target gene prediction databases and Gene database.
