# Supplementary material for: MicroRNA-34a Mediates the Aldosterone-Induced Acceleration of Endothelial Senescence
Source: Int J Hypertens. 2025 Feb 26;2025:2339598. doi: 10.1155/ijhy/2339598 (PMC11986190; doi:10.1155/ijhy/2339598)
Supplement: Supporting Information — Additional supporting information can be found online in the Supporting Information section. [file 2339598.f1.docx]

**Additional file 1: The target genes of miR-34a predicted by 10 target gene prediction databases and Gene database**

| Gene | Gene Name |
| --- | --- |
| LDHA | lactate dehydrogenase A(LDHA) |
| SLC44A2 | solute carrier family 44 member 2(SLC44A2) |
| PPP2R5A | protein phosphatase 2 regulatory subunit B'alpha(PPP2R5A) |
| JAG1 | jagged 1(JAG1) |
| VCL | vinculin(VCL) |
| CD47 | CD47 molecule(CD47) |
| WNT1 | Wnt family member 1(WNT1) |
| ARHGAP1 | Rho GTPase activating protein 1(ARHGAP1) |
| RRAS | related RAS viral (r-ras) oncogene homolog(RRAS) |
| CNTNAP1 | contactin associated protein 1(CNTNAP1) |
| MYB | MYB proto-oncogene, transcription factor(MYB) |
| PLCB1 | phospholipase C beta 1(PLCB1) |
| MYC | v-myc avian myelocytomatosis viral oncogene homolog(MYC) |
| SATB2 | SATB homeobox 2(SATB2) |
| PTPRM | protein tyrosine phosphatase, receptor type M(PTPRM) |
| FOXJ2 | forkhead box J2(FOXJ2) |
| LEF1 | lymphoid enhancer binding factor 1(LEF1) |
| DLL1 | delta like canonical Notch ligand 1(DLL1) |
| IL6R | interleukin 6 receptor(IL6R) |
| MYH9 | myosin heavy chain 9(MYH9) |
| GLCE | glucuronic acid epimerase(GLCE) |
| NUMBL | NUMB like, endocytic adaptor protein(NUMBL) |
| MYCN | v-myc avian myelocytomatosis viral oncogene neuroblastoma derived homolog(MYCN) |
| PRKD1 | protein kinase D1(PRKD1) |
| MYRIP | myosin VIIA and Rab interacting protein(MYRIP) |
| CCND1 | cyclin D1(CCND1) |
| HNF4A | hepatocyte nuclear factor 4 alpha(HNF4A) |
| VEGFA | vascular endothelial growth factor A(VEGFA) |
| PDGFRA | platelet derived growth factor receptor alpha(PDGFRA) |
| CLOCK | clock circadian regulator(CLOCK) |
| FUT8 | fucosyltransferase 8(FUT8) |
| CRLF2 | cytokine receptor-like factor 2(CRLF2) |
| DAAM1 | dishevelled associated activator of morphogenesis 1(DAAM1) |
| FKBP1B | FK506 binding protein 1B(FKBP1B) |
| PEA15 | phosphoprotein enriched in astrocytes 15(PEA15) |
| PPP1R16B | protein phosphatase 1 regulatory subunit 16B(PPP1R16B) |
| BCL2 | BCL2, apoptosis regulator(BCL2) |
| Continue |  |
|  |  |
| Gene | Gene Name |
| ACSL4 | acyl-CoA synthetase long-chain family member 4(ACSL4) |
| ELMOD1 | ELMO domain containing 1(ELMOD1) |
| ESRRA | estrogen related receptor alpha(ESRRA) |
| PODXL | podocalyxin like(PODXL) |
| MET | MET proto-oncogene, receptor tyrosine kinase(MET) |
| AXL | AXL receptor tyrosine kinase(AXL) |
| NR4A2 | nuclear receptor subfamily 4 group A member 2(NR4A2) |
| CD99 | CD99 molecule(CD99) |
| SIRT1 | sirtuin 1(SIRT1) |
| FOXP1 | forkhead box P1(FOXP1) |
| NOTCH2 | notch 2(NOTCH2) |
| NOTCH1 | notch 1(NOTCH1) |
| PLCG1 | phospholipase C gamma 1(PLCG1) |
| KLF4 | Kruppel like factor 4(KLF4) |
